# Clindamycin Derivatives: Unveiling New Prospects as Potential Antitumor Agents

**DOI:** 10.3390/ph17030276

**Published:** 2024-02-22

**Authors:** Yiduo Jia, Yinmeng Zhang, Hong Zhu

**Affiliations:** School of Chemical Engineering and Pharmacy, Wuhan Institute of Technology, Wuhan 430079, China; jiayiduo0402@163.com (Y.J.); 17871722490@163.com (Y.Z.)

**Keywords:** antitumor, MD simulation, binding force analysis, ADMET prediction, protein subcellular localization

## Abstract

This study delves into the exploration of Clindamycin derivatives, specifically compounds **3** and **3e**, to unveil their antitumor potential by employing a multidisciplinary approach. Screening a repertoire of 200 Clindamycin-associated targets pinpointed the Family A G-protein-coupled receptor as a prominent antitumor candidate. Subsequent analyses unearthed 16 pertinent antitumor proteins, with compound **3** exhibiting robust affinity towards a specific protein via stable hydrogen bonding. Molecular dynamics simulations underscored the adrenergic receptor β as a pivotal target, primarily situated in the plasma membrane and endoplasmic reticulum. These revelations hint towards compound **3**’s potential to bolster natural defense mechanisms against tumors by modulating immune responses within the tumor microenvironment, thus paving the way for novel avenues in antitumor drug development. Furthermore, employing the MTT assay, we evaluated the anti-HepG2 cell activity of compounds **3** and **3e**, with 5-fluorouracil serving as the control drug. Results revealed that compound **3** exhibited significant differences (*p* < 0.01) across all concentrations (2.5, 5, 10 μg/mL) compared to the control group, paralleled by the pronounced differences (*p* < 0.01) observed with 5-fluorouracil.

## 1. Introduction

In the protracted trajectory of pharmaceutical development, a plethora of clinical trials and protracted monitoring is typically requisite to ascertain the safety and efficacy of drugs [1]. The emergence of potential antitumor effects in established antibiotics represents a substantial opportunity to truncate the drug development timeline, potentially heralding significant breakthroughs in the field of cancer therapeutics [2].

To further elucidate this phenomenon, it becomes imperative to delve into the intricate mechanisms underlying the observed antitumor effects of antibiotics. Understanding the molecular pathways through which antibiotics exert their antineoplastic properties is paramount. Moreover, elucidating the precise mode of action can provide crucial insights into novel therapeutic targets and potential synergistic interactions with existing anticancer agents. In the context of antibiotic repurposing for cancer therapy, elucidating the pharmacokinetic and pharmacodynamic profiles assumes paramount importance. Comprehensive pharmacokinetic studies are essential to delineate the absorption, distribution, metabolism, and excretion (ADME) profiles of antibiotics in the context of cancer patients. Furthermore, meticulous pharmacodynamic assessments are necessary to ascertain the optimal dosing regimens and therapeutic windows, ensuring maximal efficacy while mitigating potential adverse effects. In addition to elucidating the mechanistic underpinnings and pharmacological profiles, clinical validation through rigorously designed clinical trials is indispensable. Well-designed prospective trials encompassing diverse patient populations are imperative to assess the safety, efficacy, and tolerability of antibiotic-based anticancer regimens. Furthermore, comparative effectiveness studies elucidating the relative merits of antibiotic-based therapies vis-à-vis conventional anticancer modalities can provide valuable insights into their clinical utility and therapeutic potential. Concurrently, it is essential to address potential challenges and limitations associated with antibiotic repurposing for cancer therapy. Foremost among these is the emergence of antibiotic resistance, which poses a significant impediment to long-term efficacy. Strategies to mitigate antibiotic resistance, such as combination therapies and targeted drug delivery systems, warrant meticulous investigation.

In conclusion, the burgeoning paradigm of antibiotic repurposing holds immense promise for revolutionizing cancer therapeutics. However, realizing this potential necessitates a multidisciplinary approach encompassing mechanistic elucidation, pharmacological characterization, and clinical validation. By surmounting these challenges, antibiotic-based anticancer therapies may emerge as indispensable tools in the oncologist’s armamentarium, ushering in a new era of precision oncology and personalized medicine.

**Figure 1:**
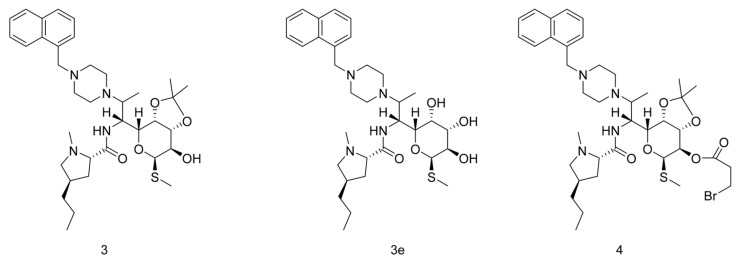
Structure of Clindamycin derivatives.

Clindamycin, recognized for its potent inhibitory activity against Gram-positive bacteria, particularly in the context of treating infectious diseases, has traditionally been considered a mainstay in microbial infection therapy. However, the exploration of its potential application in the realm of oncology has been relatively understudied in conventional perspectives. In recent investigations [3], the Intersection of Three Clindamycin Derivative Targets showed a series of Clindamycin derivatives, notably compounds **3** and **3e** in Figure 1, had demonstrated a broader spectrum of antimicrobial activity compared to conventional antibiotics. Intriguingly, these compounds exhibit significant activity not only against Gram-positive bacteria but also manifest inhibitory effects on other microbial species, underscoring their extended antimicrobial repertoire.

Of particular interest, subsequent research has unveiled that compounds **3** and **3e** display a greater array of target interactions within tumor cells compared to the targeted pathways of conventional antibiotics [4]. This revelation suggests that these compounds transcend their conventional role as antimicrobial agents and harbor notable antiproliferative activity against tumor cells [5]. The expanded scope of their target interactions implies a multifaceted mechanism of action that extends beyond the traditional paradigm of antibiotic function.

In the present study, we aim to undertake a comprehensive exploration of the antimicrobial activity of compounds **3** and **3e**, elucidating their potential mechanisms in inhibiting tumor cells. Through meticulous investigation of their cellular targets at the molecular level [6], our findings indicate a deviation from the traditional mechanisms of antibiotics, suggesting that the antiproliferative effects of these compounds may involve a more expansive array of cellular signaling pathways. This novel discovery has prompted profound contemplation regarding the potential applications of these Clindamycin derivatives in antitumor therapy, offering valuable insights for the development of innovative anticancer drugs.

During our research, we observed a substantial presence of G protein-coupled receptor (GPCR) targets among the predicted target entities [7,8]. GPCRs represent the largest integral membrane protein family in the human genome, comprising over 1000 members [9,10]. These receptors play pivotal roles in mediating signal transduction across cell membranes in response to various extracellular stimuli. Upon activation, GPCRs primarily trigger intracellular cascade reactions by interacting with heterotrimeric G proteins, although G protein-independent signaling pathways have also been described. GPCRs serve as primary conduits for information influx into cells, and their involvement is associated with numerous diseases, rendering members of this family significant pharmacological targets [11,12].

Interestingly, during the design phase of compounds **3** and **3e**, our focus was initially on targeting efflux pumps present on the cell membranes of bacteria and other microorganisms [3]. It is conceivable that targets on the microbial cell membrane and those associated with GPCRs may share structural similarities. This observation underscores the potential cross-reactivity or shared ligand-binding characteristics between the targets of compounds **3** and **3e** and those of GPCR.

Understanding the possible structural resemblances or functional overlaps between microbial membrane targets and GPCRs is crucial in assessing the pharmacological implications and off-target effects of compounds targeting efflux pumps. This insight can guide further investigations into the specific mechanisms of action and therapeutic potential of compounds **3** and **3e**, shedding light on their broader pharmacological profiles and potential applications beyond antimicrobial therapy.

The in-depth molecular analysis of compounds **3** and **3e** reveals nuanced interactions with cellular targets, suggesting a multifaceted mode of action that extends beyond conventional antibiotic paradigms. This departure from traditional mechanisms underscores the versatility of these Clindamycin derivatives, rendering them promising candidates in the pursuit of novel anticancer therapeutics. The elucidation of their differential impact on various cellular signaling pathways not only expands our understanding of their antiproliferative effects but also hints at the potential for targeted interventions in diverse cancer types.

## 2. Results

### 2.1. Intersection of Two Clindamycin Derivative Targets

**Figure 2:**
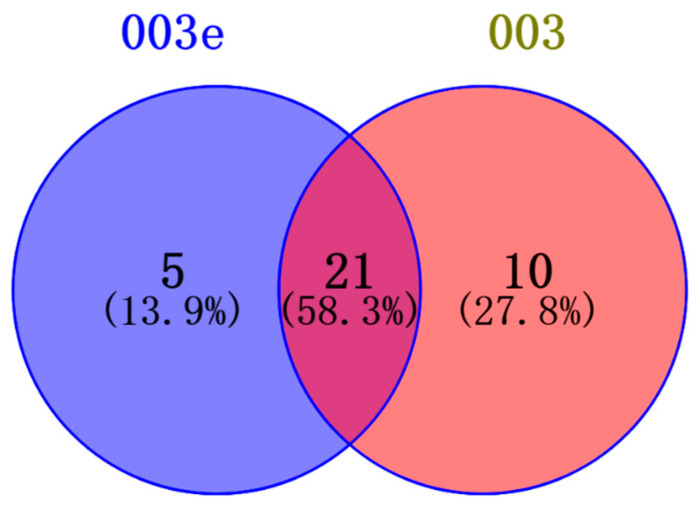
Venn diagram of targets from two compounds.

Utilizing the online software Venny 2.1.0 (csic.es) (https://bioinfogp.cnb.csic.es/tools/venny/index.html, accessed on 19 December 2023) [13], we successfully identified 21 common target points from the targets of two compounds in Figure 2.

### 2.2. Screening of the Antibacterial Targets of the Clindamycin Derivatives through the PubChem Database

Utilizing PubChem (nih.gov) (https://pubchem.ncbi.nlm.nih.gov/, accessed on 19 December 2023) [14], we identified 16 protein targets after analyzing the target organisms associated with the 21 intersecting targets. Subsequently, employing RCSBPDB (https://www.rcsb.org/, accessed on 20 December 2023) and focusing on derivatives of clarithromycin, we further screened protein targets. These selected protein targets exhibit promising antitumor properties, as indicated in Table 1.

### 2.3. Molecular Docking Simulation and Validation

We conducted a molecular docking study using the Discovery Studio 2019 client to explore the binding interactions of a specific antibiotic compound, referred to as Compound **3**. In this investigation, we considered 21 distinct protein targets as potential binding partners for both Compound **3** and Compound **3e**. To assess the reliability of the docking results, we employed the LibDock scoring system—a quantitative measure of binding affinity between molecules. Docking results with LibDock scores surpassing 100 were deemed significant, indicative of robust binding affinity, as detailed in Table 2. Subsequently, we identified and summarized protein targets with docking scores exceeding this threshold. These targets signify potential candidates influenced by the interaction and modulation of both Compound **3** and Compound **3e** [15,16].

### 2.4. Stability of the Docked Complexes Studied via MD Simulation

**Figure 3:**
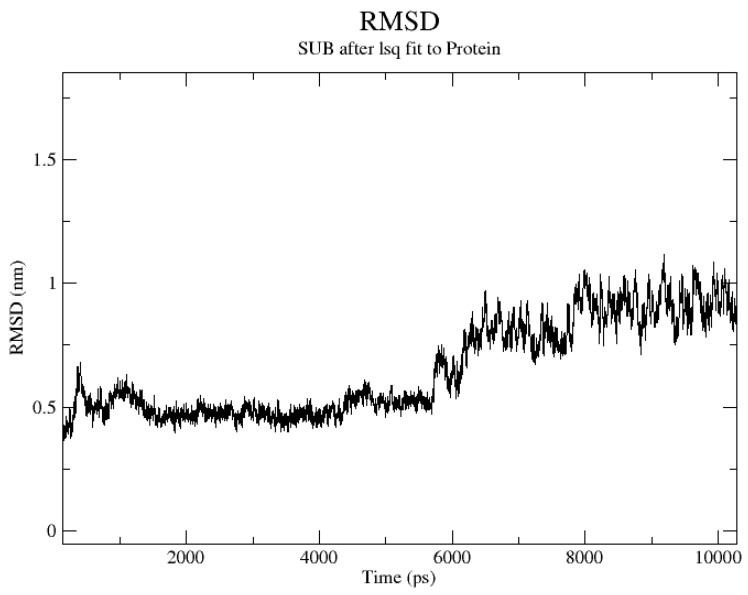
Results of the molecular dynamic simulations for four protein–compound **3** complexes. RMSD of compound **3** with protein target (PDB ID: 2rh1) in 10 ns.

**Figure 4:**
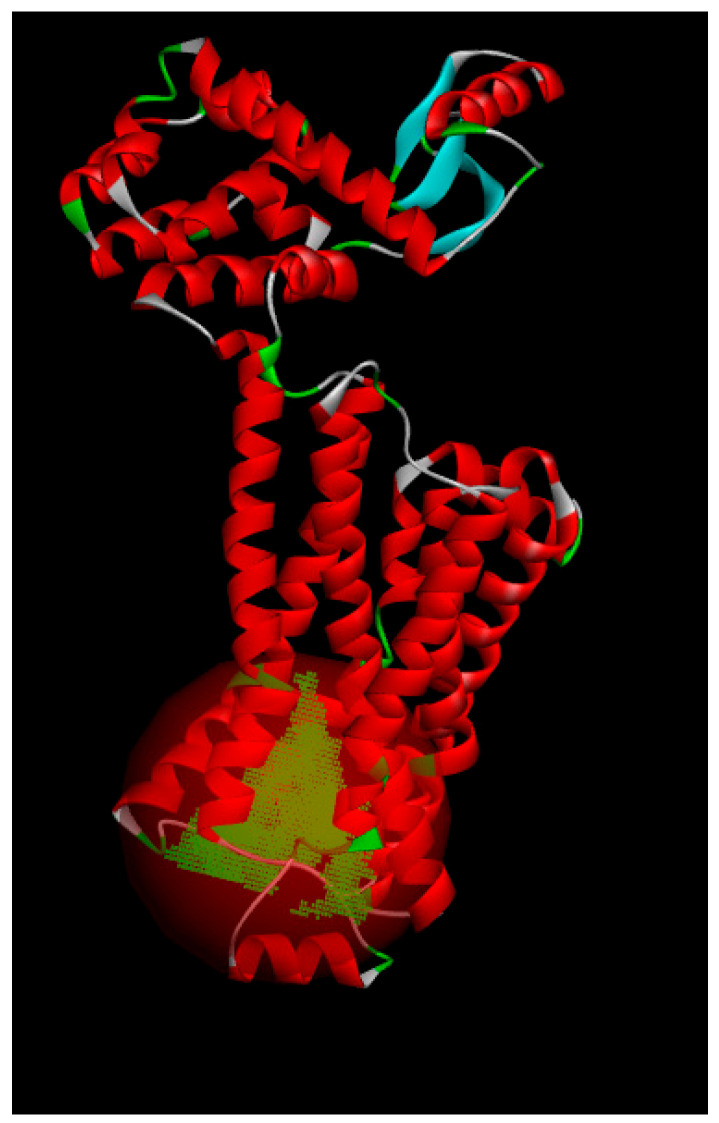
The crystal structure of the protein (PDB ID: 2rh1).

We performed in-depth conformational screening of the compound using molecular dynamics simulations. Specifically, we conducted molecular dynamics (MD) simulations involving five different proteins and compound **3** across multiple targets. Throughout these simulations, we monitored the root mean square deviation (RMSD) values for the entire system. As depicted in Figure 3, the RMSD values gradually converged during the simulation, ultimately reaching a stable state. Based on the RMSD results, we identified one protein target among the initial 21 targets that exhibited stable and rational conformational behavior. This selection was made considering the convergence and stability of the protein–ligand complex during the dynamic simulations. The crystal structure of the protein is shown in Figure 4 [17,18].

### 2.5. Binding Force Analysis

**Figure 5:**
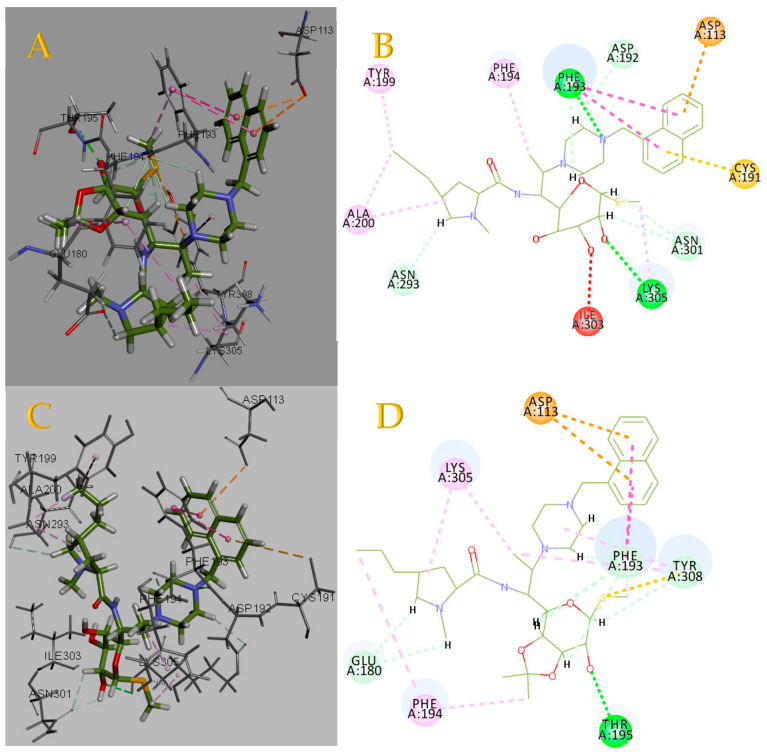
2D and 3D diagrams of the results of the docking. 2D and 3D diagrams of Compound **3** and **3e** with protein target (PDB ID: 2rh1). (**A**): 3D diagrams of Compound **3**; (**B**): 2D diagrams of Compound **3**; (**C**): 3D diagrams of Compound **3e**; (**D**): 2D diagrams of Compound **3e**.

Based on the results from the molecular dynamics (MD) simulations, we conducted a binding affinity analysis using the receptor–ligand interaction calculation tool in Discovery Studio, as illustrated in Figure 5. This analysis provided insights into the specific forces and interactions governing the binding between the compound and its target protein. The receptor–ligand interaction calculation tool in Discovery Studio allowed us to explore and characterize the key molecular interactions, such as hydrogen bonding, van der Waals forces, and electrostatic interactions, contributing to the stability and strength of the binding between the compound and the protein target [19,20].

The interactions between protein (PDB ID: 2rh1) and compound **3** mainly comprised hydrogen bonds, electrostatic and hydrophobic interactions, and miscellaneous, as depicted in Figure 5A. For the hydrogen bonds, residue 195 formed one conventional hydrogen bond with a bond length of 2.08 Å. Residue 180, residue 193, and residue 308 formed three carbon–hydrogen bonds. For electrostatic interactions, residue 113 formed one pi–cation bond. For the hydrophobic interactions, residues193, 194, and 305 formed one pi–pi staked bond, one pi–alkyl bond, and one alkyl bond.

The interactions between protein (PDB ID: 2rh1) and compound **3e** mainly comprised hydrogen bonds, electrostatic and hydrophobic interactions, and miscellaneous, as shown in Figure 5B. For hydrogen bonds, residues 193 and 305 formed one conventional hydrogen bond with bond lengths of 2.95 Å and 2.12 Å. Residue 192 and residue 293 formed two carbon–hydrogen bonds. For electrostatic interactions, residue 113 formed one pi–cation bond. For the hydrophobic interactions, residues193, 194, and 305 formed one pi–pi staked bond, one pi–alkyl bond, and one alkyl bond.

The results from Table 3 indicate that compound **3** exhibits a Gibbs free energy of −23.8668 and a dissociation constant of 1108.10, as estimated via molecular docking. These values suggest a strong affinity and stability of compound **3** towards the 2RH1 protein compared to compound **3e**.

A lower Gibbs free energy indicates a more favorable and stable binding interaction between the compound and the protein. In this case, the significantly negative value of the Gibbs free energy for compound **3** suggests a highly stable interaction with the 2RH1 protein [21].

Moreover, the dissociation constant is inversely related to the binding affinity, with lower values indicating stronger binding. The relatively high dissociation constant of 1108.10 for compound **3** indicates a low dissociation rate and high stability of the compound–protein complex. This suggests that compound **3** has a strong tendency to remain bound to the 2RH1 protein, implying a favorable and robust binding interaction [22,23].

Comparatively, the observed higher affinity and stability of compound **3** towards the 2RH1 protein, as indicated through its lower Gibbs free energy and higher dissociation constant, highlight its potential as a promising ligand or drug candidate for targeting the specific binding site on the protein. The distinction between compound **3** and compound **3e** lies in the presence of hydroxyl groups at positions 3 and 4. Upon alkylating the two hydroxyl groups in compound **3e**, the inhibitory effect of compound **3** on residue 303 is abolished. Consequently, the entire molecule exhibits enhanced antitumor efficacy, particularly evident in its activity against tumor cells.

This observation underscores the critical role of structural modifications in optimizing the pharmacological properties of compounds. By selectively alkylating the hydroxyl groups, compound **3e** circumvents the inhibitory effects on residue 303, resulting in improved antitumor activity. These findings underscore the potential of compound **3e** as a promising candidate for anticancer therapy, emphasizing the importance of molecular structural optimization in achieving desirable therapeutic outcomes.

### 2.6. Protein Subcellular Localization

Based on molecular docking and molecular dynamics simulations, we identified the Adrenergic receptor beta as a crucial protein target. Subsequently, we conducted a subcellular localization analysis based on the amino acid sequences of these proteins. In Table 4, our findings reveal that Adrenergic receptor beta predominantly localizes to the plasma membrane (52.2%). Additionally, a significant proportion is observed in the endoplasmic reticulum (26.1%). These results shed light on the predominant subcellular localization of Adrenergic receptor beta, providing valuable insights for further exploration of its biological functions [24].

### 2.7. Biological Evaluation of Compounds

**Figure 6:**
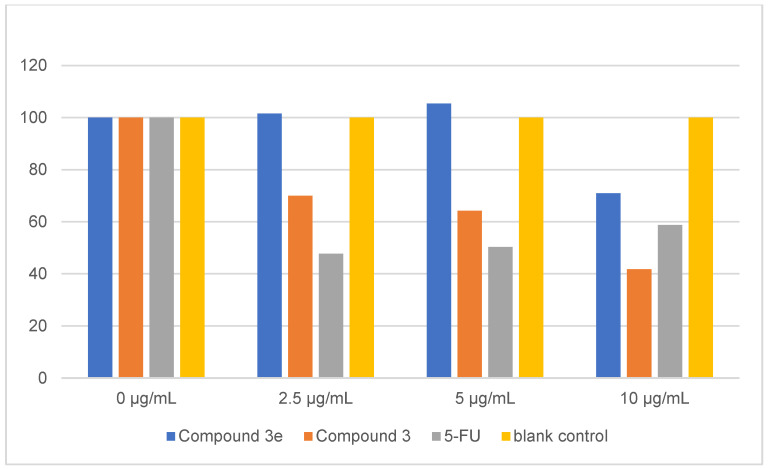
Toxicity of compounds.

The synthesized compounds were investigated for their potential to impede the proliferation of HepG2 cells [25,26]. Analysis of the data presented in Table 5 and Figure 6 reveals that both compound **3** and compound **3e** significantly reduce cell viability in HepG2 cells after 24 h, exhibiting a concentration-dependent relationship where higher concentrations correlate with decreased cell survival rates. In comparison to the control group, compound **3e** demonstrates statistically significant differences (*p* < 0.01) in cell viability only at concentrations of 5 and 10 μg/mL. Conversely, compound **3** displays significant disparities (*p* < 0.01) across all tested concentrations relative to the control group. Furthermore, 5-fluorouracil (5-FU) exhibits pronounced discrepancies (*p* < 0.01) compared to the control group. These results collectively suggest that these compound classes exert a significant inhibitory effect on the growth of HepG2 cells [27,28].

## 3. Materials and Methods

### 3.1. Intersection of Two Clindamycin Derivative Targets

Upon scrutinizing the literature, it has come to our attention that compound **4** in Figure 1 exhibits a limited number of identified antitumor targets. Simultaneously, leveraging the structural features of compounds **3** and **3e**, we hypothesize their potential in vitro antitumor activity. To gain deeper insights into the target proteins associated with these compounds, we employed the online tool (https://bioinfogp.cnb.csic.es/tools/venny/index.html, accessed on 20 December 2023) to intersect the predicted top 100 target proteins of compound **3** and compound **3e**. This intersection revealed a set of common target proteins.

### 3.2. Screening of the Antibacterial Targets of the Clindamycin Derivatives through the PubChem Database

To further refine target selection and investigate the interactions between these targets, we systematically screened 21 protein targets using the PubChem database (https://pubchem.ncbi.nlm.nih.gov/, accessed on 20 December 2023). The selection criteria were based on the keyword search “anti-tumor, Homo sapiens”, aimed at identifying protein targets associated with anticancer properties. This approach is instrumental in narrowing down potential candidates for in-depth investigation, providing a foundation for elucidating the molecular mechanisms underlying the antitumor effects of the compounds under study.

### 3.3. Molecular Docking Simulation and Validation

In this process, we utilized Discovery Studio 2019 Client to construct a ligand library and performed docking studies with CHARMM, refining the ligand shapes and charge distributions. This allowed us to analyze the binding interactions between Clindamycin derivatives and drug targets. Employing LibDock scores, we systematically selected optimal binding poses, filtering out targets with scores less than 100. This rigorous approach has provided valuable insights into the binding mechanisms, enhancing our understanding of the interaction dynamics between the Clindamycin derivatives and the selected drug targets.

### 3.4. Stability of the Docked Complexes Studied via MD Simulation

Molecular dynamics (MD) simulations utilizing GROMACS (version 2020.3) were employed to scrutinize the dynamics of protein–ligand binding. Protein structures were refined using the AMBER99SB-ILDN force field, and water molecules were represented employing the TIP3P model. ACPYPE was enlisted for ligand charge calculations and the generation of GAFF force-field-compatible files. Simulations were set up within cubic boxes, ensuring a minimum atom-box boundary distance of 0.8 nm, and hydrated with SOL water at a density of 1000 g/L. To maintain electrical neutrality, chloride ions were introduced, replacing solvent water. An initial energy minimization step was conducted to relax the system, succeeded by a 100 ps restrained MD simulation at 298.15 K. Unrestricted MD simulations with a time step of 0.002 ps were subsequently carried out for 10 ns while preserving isothermal–isobaric conditions at 298.15 K and 1 bar pressure. The control of these conditions was facilitated using thermostats and barostats.

### 3.5. Binding Force Analysis

The estimation of binding free energy in protein–ligand complexes was conducted using the MM/PBSA equation. The APBS lattice parameters were determined based on MD results, and the APBS module within Discovery Studio 2019 Client (Pacific Northwest National Laboratory, Richland, DC, USA) was utilized to calculate polar solvation energy (PB) and nonpolar solvation energy (SA). The binding free energy (∆G_Bind) is calculated according to the following equation:

∆G_Bind = ∆G_Complex − ∆G_Ligand − ∆G_Receptor.

The MM energy encompasses intermolecular interactions, such as van der Waals forces, electrostatic interactions, and hydrogen bonding, between the protein and the ligand. This energy is typically computed using a force field that approximates the potential energy function of the system. Polar solvation energy (∆G_PB) characterizes the interaction between solvent molecules (typically water molecules) and polar atoms (partially charged) in proteins and ligands. Nonpolar solvation energy (∆G_SA) describes the interaction between solvent molecules and nonpolar regions (typically hydrophobic hydrocarbon chains) in proteins and ligands.

In this study, we employed molecular simulation techniques to investigate the noncovalent binding processes of compound **3** and compound **3e** with the active site 90 of the 2RH1 protein. Molecular docking was conducted using Discover Studio software (21.1.0.20298), with the CHARMM force field and the Poisson–Boltzmann with nonpolar Surface Area (PBSA) solvent model employed for accurate description of molecular interactions and solvent effects.

The Gibbs free energy (Δ*Gdis*) is considered a critical indicator for assessing the stability of interactions between compounds and proteins. To further understand the nature of these interactions, the dissociation constant (*Kdis*) was calculated using the following equation:

ΔGdis=−RT ln Kdis.

Here, Δ*Gdis* represents the change in Gibbs free energy during the dissociation process, while *R* denotes the gas constant, and *T* represents the absolute temperature. The dissociation constant, *Kdis*, describes the stability of binding between the compounds and the protein, with lower values indicating greater stability.

### 3.6. Protein Subcellular Localization

Subcellular localization refers to the specific compartment or organelle within a cell where a protein is located. It plays a crucial role in understanding the function and regulatory mechanisms of proteins within the complex cellular environment. Various experimental and computational methods have been developed to predict the subcellular localization of proteins, and PSORT is one such computer program designed for this purpose.

PSORT (Protein Subcellular Localization Prediction Tool) is a bioinformatics tool utilized to predict the subcellular localization of proteins. It primarily relies on the analysis of amino acid sequences and additional source information to make predictions about where a given protein is likely to be localized within a cell. To predict subcellular localization, the amino acid sequences of target proteins are obtained from the UniProt database, and these sequences are then inputted into the PSORT II online software (https://psort.hgc.jp/, accessed on 21 December 2023). PSORT II provides predictions regarding the subcellular location of these target proteins.

### 3.7. Biological Evaluation of Compounds

According to the simulated results, we conducted in vitro anti-HepG2 tumor cell activity tests using the MTT assay for compound **3** and compound **3e**, with 5-fluorouracil serving as the positive control. Log-phase cells were harvested and adjusted to a density of 9 × 10^4^ cells/mL and then gently mixed before seeding into a 96-well plate at 100 μL per well, with 150 μL of PBS solution added to the edge wells for humidity preservation. After 24 h of cell adhesion, the cell supernatant was discarded, and 100 μL of different compound DMSO solutions (compound **3**, compound **3e**, and 5-fluorouracil) at final concentrations of 2.5, 5, and 10 μg/mL were added to each well. Normal control wells (cultured in DMEM medium) and positive control wells (5-fluorouracil at concentrations of 10 μg/mL and 2.5 μg/mL) were also set up, with 3 replicate wells per concentration.

Following 24 h of incubation in a CO_2_ incubator, the original culture medium was aspirated, and each well was washed with 100 μL of PBS. Subsequently, 100 μL of 1 mg/mL MTT solution was added to each well and incubated for 4 h in the CO_2_ incubator. After discarding the MTT solution, 150 μL of DMSO solution was added to each well, followed by gentle shaking for 10 min. The absorbance (A) value was measured at 490 nm using an enzyme-labeling instrument, and the cell survival rate was calculated using the formula: Cell survival rate = (Aa − Ac)/(Ab − Ac) × 100%, where Aa represents the absorbance of the experimental well, Ab represents the absorbance of the normal control well, and Ac represents the absorbance of the blank well.

All data were analyzed using SPSS 23.0 statistical software, with results presented as ±s. Intergroup comparisons were conducted using one-way analysis of variance, and statistical significance was determined at *p* < 0.05 or *p* < 0.01.

## 4. Conclusions

In conclusion, our study highlights the potential of compounds **3** and **3e** as effective antitumor agents, targeting a broad range of cellular pathways associated with tumor suppression. Through structural modifications, compound **3e** exhibits enhanced antitumor efficacy compared to compound **3** by circumventing inhibitory effects on residue 303. Molecular docking and dynamics simulations identify the adrenergic receptor β as a pivotal protein target, suggesting a role in regulating immune responses within the tumor microenvironment. These findings offer valuable insights for the development of novel antitumor drugs, emphasizing the importance of structural optimization in achieving desirable therapeutic outcomes.

## Figures and Tables

**Table 1:** Results of screened targets via PubChem database.

Target	Common Name	PDB ID	Uniprot ID	ChEMBL ID	Target Class
Serotonin 1a (5-HT1a) receptor	HTR1A	7E2X	P08908	CHEMBL214	Family A G protein-coupled receptor
C-C chemokine receptor type 1	CCR1	7VL8	P32246	CHEMBL2413	
Nociceptin receptor	OPRL1	4EA3	P41146	CHEMBL2014	
Serotonin 2a (5-HT2a) receptor	HTR2A	6WHA, 7VOD, 6A93, 6WGT	P28223	CHEMBL224	
Alpha-1a adrenergic receptor	ADRA1A	8THK	P35348	CHEMBL229	
Histamine H1 receptor	HRH1	3RZE	P35367	CHEMBL231	
C-C chemokine receptor type 3	CCR3	4ZYA, 5XIX	P51677	CHEMBL3473	
Serotonin 1d (5-HT1d) receptor	HTR1D	7E32	P28221	CHEMBL1983	
Serotonin 1b (5-HT1b) receptor	HTR1B	4IAQ	P28222	CHEMBL1898	
Adrenergic receptor beta	ADRB2	2RH1	P07550	CHEMBL210	
Serotonin 4 (5-HT4) receptor	HTR4	7XT8	Q13639	CHEMBL1875	
Alpha-2a adrenergic receptor	ADRA2A	7W7E	P08913	CHEMBL1867	

**Table 2:** Lib Dock Score of Docking.

PDB ID	Lib Dock Score	
	3	3e
7VL8	127.398	126.845
4EA3	152.611	149.226
7VOD	143.659	159.64
6WHA	143.333	157.689
8THK	157.21	160.324
5XIX	107.145	127.742
7E32	118.02	115.01
6A93	106.887	159.495
4IAQ	155.758	159.28
2RH1	164.678	184.081

**Table 3:** Gibbs free energy values ΔG_dis_ (kcal.mol^−1^) and Dissociation constant K_dis_ (µM) of 2RH1.

Compound	Gibbs Free Energy Values	Dissociation Constant
**3**	−23.8668	1108.10
**3e**	−6.2632	28.57

**Table 4:** Results of Docking.

Drug	Protein	Location (k = 23)	PDB ID
Compound **3**	Adrenergic receptor beta	52.2%: plasma membrane	2RH1
		26.1%: endoplasmic reticulum	
		8.7%: vacuolar	
		4.3%: mitochondrial	
		4.3%: nuclear	
		4.3%: Golgi	

**Table 5:** Toxicity of compound **3**, compound **3e** and **5**-FU (x ± s, *n* = 3).

Drug	0 μg/mL	2.5 μg/mL	5 μg/mL	10 μg/mL
Compound **3e**	100 ± 0.05	101.57 ± 2.48	105.41 ± 1.53 **	70.95 ± 2.60 **
Compound **3**	100 ± 0.05	69.98 ± 3.51 **	64.26 ± 1.18 **	41.75 ± 3.27 **
5-FU	100 ± 0.05	47.72 ± 3.59 **	50.28 ± 3.87 **	58.78 ± 6.97 **
blank control	100 ± 0.05	100 ± 0.05	100 ± 0.05	100 ± 0.05

**: Compared to the blank control group, *p* < 0.01.

## Data Availability

Data is contained within the article.

## References

[1] Robinson P.C., Liew D.F., Tanner H.L., Grainger J.R., Dwek R.A., Reisler R.B., Steinman L., Feldmann M., Ho L.-P., Hussell O.S. (2022). COVID-19 therapeutics: Challenges and directions for the future. Proc. Natl. Acad. Sci. USA.

[2] Wang M., Rousseau B., Qiu K., Huang G., Zhang Y., Su H., Le Bihan-Benjamin C., Khati I., Artz O., Foote M.B. (2023). Killing tumor-associated bacteria with a liposomal antibiotic generates neoantigens that induce anti-tumor immune responses. Nat. Biotechnol..

[3] Jia Y., Zhang Y., Zhu H. (2023). Structure–Activity Relationship Target Prediction Studies of Clindamycin Derivatives with Broad-Spectrum Bacteriostatic Antibacterial Properties. Molecules.

[4] Childs-Disney J.L., Yang X., Gibaut Q.M., Tong Y., Batey R.T., Disney M.D. (2022). Targeting RNA structures with small molecules. Nat. Rev. Drug Discov..

[5] Adewumi O.A., Singh V., Singh G.J. (2020). Phytochemistry Chemical composition, traditional uses and biological activities of artemisia species. J. Pharmacogn. Phytochem..

[6] Damavandi M.S., Shojaei H., Esfahani B.N. (2023). The anticancer and antibacterial potential of bioactive secondary metabolites derived from bacterial endophytes in association with *Artemisia absinthium*. Sci. Rep..

[7] Liu J., Tang H., Xu C., Zhou S., Zhu X., Li Y., Prézeau L., Xu T., Pin J.-P., Rondard P.J. (2022). Biased signaling due to oligomerization of the G protein-coupled platelet-activating factor receptor. Nat. Commun..

[8] Yang D., Zhou Q., Labroska V., Qin S., Darbalaei S., Wu Y., Yuliantie E., Xie L., Tao H., Cheng J.J. (2021). G protein-coupled receptors: Structure-and function-based drug discovery. Signal Transduct. Target. Ther..

[9] Leon K., Cunningham R.L., Riback J.A., Feldman E., Li J., Sosnick T.R., Zhao M., Monk K.R., Araç D.J. (2020). Structural basis for adhesion G protein-coupled receptor Gpr126 function. Nat. Commun..

[10] Sandhu M., Cho A., Ma N., Mukhaleva E., Namkung Y., Lee S., Ghosh S., Lee J.H., Gloriam D.E., Laporte S.A. (2022). Dynamic spatiotemporal determinants modulate GPCR: G protein coupling selectivity and promiscuity. Nat. Commun..

[11] Benemei S. (2022). Molecular Agonists Mechanisms of 5-HT1F Receptor. Novel Synthetic Drugs in Migraine.

[12] Mir M.A. (2023). Cytokine and Chemokine Networks in Cancer.

[13] Zhuge H., Ge Z., Wang J., Yao J., He J., Wang Y., Wang Y., Tang Y. (2023). The Tandem of Liquid Chromatography and Network Pharmacology for the Chemical Profiling of Pule’an Tablets and the Prediction of Mechanism of Action in Treating Prostatitis. Pharmaceuticals.

[14] Praveen M., Ullah I., Buendia R., Khan I.A., Sayed M.G., Kabir R., Bhat M.A., Yaseen M. (2024). Exploring *Potentilla nepalensis* Phytoconstituents: Integrated Strategies of Network Pharmacology, Molecular Docking, Dynamic Simulations, and MMGBSA Analysis for Cancer Therapeutic Targets Discovery. Pharmaceuticals.

[15] Keretsu S., Bhujbal S.P., Cho S.J. (2020). Rational approach toward COVID-19 main protease inhibitors via molecular docking, molecular dynamics simulation and free energy calculation. Sci. Rep..

[16] Opo F.A.D.M., Rahman M.M., Ahammad F., Ahmed I., Bhuiyan M.A., Asiri A.M. (2021). Structure based pharmacophore modeling, virtual screening, molecular docking and ADMET approaches for identification of natural anti-cancer agents targeting XIAP protein. Sci. Rep..

[17] Fu H., Chen H., Blazhynska M., Goulard Coderc De Lacam E., Szczepaniak F., Pavlova A., Shao X., Gumbart J.C., Dehez F., Roux B. (2022). Accurate determination of protein: Ligand standard binding free energies from molecular dynamics simulations. Nat. Protoc..

[18] Zeng Z., Wodaczek F., Liu K., Stein F., Hutter J., Chen J., Cheng B. (2023). Mechanistic insight on water dissociation on pristine low-index TiO_2_ surfaces from machine learning molecular dynamics simulations. Nat. Commun..

[19] Almontasser A., Al-Mufti S.M., Arya R.K. (2023). Application of Nanofillers in Drug Delivery Industry. Handbook of Nanofillers.

[20] Wang H., Yang Y., Zeng Y., Li L. (2023). Ionic Liquid-Based Sensors for Fast Determination of Aromatic Compounds in the Environment. Encyclopedia of Ionic Liquids.

[21] Robo M.T., Hayes R.L., Ding X., Pulawski B., Vilseck J.Z. (2023). Fast free energy estimates from λ-dynamics with bias-updated Gibbs sampling. Nat. Commun..

[22] Liu W., Chen L., Yin D., Yang Z., Feng J., Sun Q., Lai L., Guo X. (2023). Visualizing single-molecule conformational transition and binding dynamics of intrinsically disordered proteins. Nat. Commun..

[23] Kruse M., Altattan B., Laux E.-M., Grasse N., Heinig L., Möser C., Smith D.M., Hölzel R. (2022). Characterization of binding interactions of SARS-CoV-2 spike protein and DNA-peptide nanostructures. Sci. Rep..

[24] Kobayashi H., Cheveralls K.C., Leonetti M.D., Royer L.A. (2022). Self-supervised deep learning encodes high-resolution features of protein subcellular localization. Nat. Methods.

[25] Yu Y., Lin R., Yu H., Liu M., Xing E., Wang W., Zhang F., Zhao D., Li X. (2023). Versatile synthesis of metal-compound based mesoporous Janus nanoparticles. Nat. Commun..

[26] Mehany M.M., Hammam O.A., Selim A.A., Sayed G.H., Anwer K.E. (2024). Novel pyridine bearing pentose moiety-based anticancer agents: Design, synthesis, radioiodination and bioassessments. Sci. Rep..

[27] Sroor F.M., Mahrous K.F., El-Kader H.A.M.A., Othman A.M., Ibrahim N.S. (2023). Impact of trifluoromethyl and sulfonyl groups on the biological activity of novel aryl-urea derivatives: Synthesis, in-vitro, in-silico and SAR studies. Sci. Rep..

[28] El-Fakharany Z.S., Nissan Y.M., Sedky N.K., Arafa R.K., Abou-Seri S.M. (2023). New proapoptotic chemotherapeutic agents based on the quinolone-3-carboxamide scaffold acting by VEGFR-2 inhibition. Sci. Rep..

